# Racism against healthcare users in inpatient care: a scoping review

**DOI:** 10.1186/s12939-024-02156-w

**Published:** 2024-05-02

**Authors:** Sibille Merz, Tuğba Aksakal, Ariam Hibtay, Hilâl Yücesoy, Jana Fieselmann, Kübra Annaç, Yüce Yılmaz-Aslan, Patrick Brzoska, Hürrem Tezcan-Güntekin

**Affiliations:** 1 Faculty of Health and Education, Alice Salomon University of Applied Sciences, Alice-Salomon-Platz 5, 12627 Berlin, Germany; 2 Faculty of Health, School of Medicine, Witten/Herdecke University, Health Services Research Unit. Alfred-Herrhausen-Straße 50, 58448 Witten, Germany

**Keywords:** Racism, Healthcare, Inpatient, Hospital, Rehabilitation, Intersectional, High-income countries

## Abstract

**Background:**

Racism in the healthcare system has become a burgeoning focus in health policy-making and research. Existing research has shown both interpersonal and structural forms of racism limiting access to quality healthcare for racialised healthcare users. Nevertheless, little is known about the specifics of racism in the inpatient sector, specifically hospitals and rehabilitation facilities. The aim of this scoping review is therefore to map the evidence on racial discrimination experienced by people receiving treatment in inpatient settings (hospitals and rehabilitation facilities) or their caregivers in high-income countries, focusing specifically on whether intersectional axes of discrimination have been taken into account when describing these experiences.

**Methods:**

Based on the conceptual framework developed by Arksey and O’Malley, this scoping review surveyed existing research on racism and racial discrimination in inpatient care in high-income countries published between 2013 and 2023. The software Rayyan was used to support the screening process while MAXQDA was used for thematic coding.

**Results:**

Forty-seven articles were included in this review. Specifics of the inpatient sector included different hospitalisation, admission and referral rates within and across hospitals; the threat of racial discrimination from other healthcare users; and the spatial segregation of healthcare users according to ethnic, religious or racialised criteria. While most articles described some interactions between race and other social categories in the sample composition, the framework of intersectionality was rarely considered explicitly during analysis.

**Discussion:**

While the USA continue to predominate in discussions, other high-income countries including Canada, Australia and the UK also examine racism in their own healthcare systems. Absent from the literature are studies from a wider range of European countries as well as of racialised and disadvantaged groups other than refugees or recent immigrants. Research in this area would also benefit from an engagement with approaches to intersectionality in public health to produce a more nuanced understanding of the interactions of racism with other axes of discrimination. As inpatient care exhibits a range of specific structures, future research and policy-making ought to consider these specifics to develop targeted interventions, including training for non-clinical staff and robust, transparent and accessible complaint procedures.

## Background

Racism has long been recognised as a social determinant of health [1, 2]. In addition to the different health effects of racial discrimination, a burgeoning focus in health policy-making and research has been the impact of racism in the healthcare system. Existing evidence has demonstrated the extent to which racialised healthcare users, and often staff, are exposed to racism in healthcare-related encounters [3, 4]. Two primary manifestations of racism in healthcare have consistently been identified: implicit bias in interpersonal encounters between healthcare providers and users, and structural forms of discrimination.

Implicit bias by healthcare providers, especially physicians [5–7] has been found to be pervasive. A study from the USA, for instance, found that the racist assumptions of African Americans’ higher threshold for pain has led to their systematic undertreatment [8]. Similarly, a study of ethnic and racial discrimination among US veterans with pain identified a high level of dissatisfaction with regard to interactions with staff for Latinx patients, and with negative demeanour of staff for African American patients [9]. Implicit bias can also lead to differential treatment and diagnoses. For example, African Americans in forensic psychiatric hospitals are disproportionately more often diagnosed with highly stigmatised psychotic spectrum disorders as compared to white Americans [10].

Discrimination can have detrimental effects on patients’ decision-making capabilities and their trust towards physicians [9, 11]. It can negatively shape the doctor-patient relationship and patients’ satisfaction with healthcare delivery [11], which may in turn impact patients’ adherence to and engagement with treatment and thus ultimately exacerbate health inequities [11]. Indeed, a review of the perspectives of health professionals and patients on racism in healthcare has confirmed that implicit bias can lead to the further alienation of minoritised individuals from the public healthcare system as a whole [4]. This is especially the case as healthcare provider bias is often not acknowledged [12].

At the same time, existing research has found structural barriers to accessing care. A particular focus has been on barriers due to limited language skills [13–15]. For instance, a study from the US context [16] examined the use of evidence-based healthcare services for chronic disease management by both Latinx and white patients. They found that white patients were more likely than Latinxs to access the recommended services for which they were eligible; however, when grouping study participants by language use, they found that English-speaking Latinxs were not significantly less likely to access those services. This was in stark contrast to those Latinx patients who spoke Spanish at home and were least likely to access the recommended services even after accounting for possible confounding variables. A similar study from the German context [14] determined that the lack of interpreters and the dearth of multilingual information constitutes a form of institutional or structural racism which can negatively affect patients’ communication, diagnostic procedures and treatment options. This can ultimately lead to the systematic disadvantage of migrants and their descendants, minimising their chances for access to and use of health-related services [17]. While this knowledge is crucial to addressing existing inequities, a limitation of existing research is that the focus is predominantly on recent immigrants and their families, equating racism with xenophobia and migration, and leaving unaddressed the multiple and interlocking forms of discrimination which are independent of citizenship and settlement status. Indeed, the focus on linguistic barriers, while important, misses the fact that racialised people experience racism independent of their linguistic capabilities, mother tongue or migration status.

A range of reviews and meta-analyses have been conducted to examine the nature and scope of racial discrimination in the healthcare system. While some have focused on specific aspects, for example implicit bias [5, 6, 18], anti-racist interventions [19] or public health understandings of structural racism [20], others have been more comprehensive [3]. These reviews have substantially contributed to the understanding of how racism operates in healthcare interactions, and the forms it can take. However, three dimensions appear to have only been marginally addressed.

First, most existing research has focused on outpatient services or has not been specific about the examined setting. This might be due to the overriding focus on implicit racial biases and their effects on the doctor-patient relationship, which is applicable across settings. However, we believe it is crucial to study the inpatient setting in more detail. This is, first, because there is mounting evidence that the work-related stress associated with inpatient care may fuel the stereotyping of patients and amplify discrimination [21]. Second, patients treated in inpatient settings might have different disease profiles and adhere to different treatment regimens, which might create diverging discriminatory practices and structures. Not least, the experiences of racism might differ in inpatient, enclosed settings where the option of leaving and choosing another provider might simply not exist. As such, it is critical to develop a deeper and more comprehensive understanding of how racism pans out in inpatient care, and the characteristics of this setting that are amenable to, or even fuel, discrimination.

Second, the majority of existing research has examined the US context, especially discrimination against African Americans. This may well be due to the country’s history of transatlantic enslavement and racial violence [22], persistent anti-Black racism [23] as well as the size and scope of anti-racist scholarship across disciplines in the US. Not least, it may be the result of the availability of data segregated by racial/ethnic groups as mandated by the National Institutes of Health for clinical research [24]. However, the focus on the unique dynamics and historical context of the US encapsulates only a small part of how racism may manifest across different cultural and national contexts.

Third, we found that few existing reviews have thoroughly engaged with how intersectional perspectives have been adopted in the studies they analysed (e.g. [3]), or examined how the entanglements of race with other social categories such as gender or socio-economic status affect the experience of racism in healthcare. This, however, is crucial, as it has long been known that the intersections of multiple axes of discrimination not only produce layered but also unique experiences and health inequities [25, 26]. For example, it has been found that Black women have a higher risk of poor cardiovascular health compared to both Black men and white women [27]. This health outcome would have remained invisible using a single-category approach focusing either on race or on gender. As such, we aim to assess specifically how experiences of racism in interaction with sexism and other axes of discrimination have been described in the literature on racism in inpatient care.

The aim of this scoping review is therefore to map the evidence on racial discrimination experienced by people receiving treatment in inpatient settings (hospitals and rehabilitation facilities) or their caregivers in high-income countries, defined as having a gross national income per capita of $12,736 or more using the World Bank Atlas method [28]. Most high-income countries share colonial histories on which their wealth has been built such that their modern-day societies are shot through with racist, racialised and racialising structures and imaginaries [22, 29]. Naturally, they also significantly differ in terms of their precise historical trajectories and present-day effects, especially between former settler colonial states and other forms of colonial domination [30]. Sometimes postcolonial states or societies also have closer ties and more similarities with their former colonisers than former colonisers with each other, for instance a common cultural heritage, linguistic practices and political models [31, 32]. The exclusive focus on high-income countries is therefore somewhat arbitrary but borne of the need for feasibility. We thereby address specifically whether intersectional axes of discrimination have been taken into account, and, if yes, how so, when describing such experiences of racism. The results of this review will be utilised further to develop data collection instruments (surveys, topic guides for qualitative interviews) on experiences of racism in the German healthcare system.

This scoping review is part of a larger research project that sets out to empirically investigate experiences, situations, and interpretations of racism and racial discrimination in healthcare institutions in Germany, specifically in the inpatient sector. The project builds on the theoretical conceptualisations of everyday racism following Essed [33] and Terkessidis [34, 35]. Everyday racism is the often subtle but pervasive form of racism encountered by racialised people in routine everyday life. Essed thereby distinguishes between macro or structural-cultural properties of racism, and more interactional or interpersonal forms, which she refers to as “micro-inequities perpetuating the system” (1991: 38). The macro properties of racism encompass the racism engrained in government agencies, businesses and organisations responsible for legislation and policy-making; its effects can therefore be seen in labour policy, healthcare, education, or housing [33]. An example from the healthcare context might be the structurally entrenched lack of interpreters and multilingual and culturally appropriate information [14, 36] or the unequal spatial distribution of health services [37]. White-Means and Muruako (2023), for instance, found that metropolitan areas with a majority of spatially segregated, low-income and Black households are more likely to have disparities in access to primary breast cancer care than middle-income Black neighbourhoods or middle-income white neighbourhoods [37]. Examples of the everyday perpetuation of racism through individual practices include the implicit racial bias by healthcare providers [6], often leading to mistrust by people having experienced unequal treatment as a result of such bias [38]. However, Essed aptly stresses that such forms of racism are not the result of individual bias but rather the ways in which “the system is continually construed in everyday life” (1991: 38). In this context, it is worth pointing out that the explicit use of the terms race and racism are relatively rare in Europe as compared to the US. Many European countries use the euphemisms ‘ethnicity’ or ‘migration background’, themselves the result of colonial histories and racist labour policies [39], when describing racialised groups and the discrimination they experience. While such designations may not use purely biological but often cultural markers, they have become racialised as they are often regarded as unitary groups based on shared cultural traits [40]. As such, using analytical approaches focusing on race and racism is highly productive.

In addition, in this review we draw on intersectionality scholarship, which focuses on the intersectional effects of multiple forms of discrimination and regimes of power [41]. The concept of intersectionality has become increasingly popular in public health as it provides a lens to identify the unique and complex health inequities faced by groups at multiple axes of discrimination [42, 43]. An intersectional approach thus allows not only for a more detailed understanding of health inequities, but also for the development of more targeted public health interventions to reduce these.

## Methods

### Framework

This review used the conceptual framework developed by Arksey and O’Malley [44] to guide its methodology. This framework consists of the following five stages: (1) identifying the research question; (2) identifying relevant studies; (3) study selection; (4) charting the data; and (5) collating, summarising, and reporting the results. The study protocol has been registered on Open Science Frameworks (https://osf.io/xaz2s).

### Stage 1: identifying the research question

The aim of this scoping review was to map the evidence on racial discrimination by people receiving treatment in inpatient settings (hospitals and rehabilitation facilities) or their caregivers in high-income countries. In particular, we aimed to assess whether, and if so how, intersectional axes of discrimination had been taken into account when describing these experiences. This interest stems from our earlier work in migration studies, analysing the health needs of migrant communities, as well as intersectional and diversity-sensitive health interventions [45–47]. Not least, as some of us have experienced structural or interpersonal racism first hand or witnessed such racism against family members, while others are allies, we aimed to develop these research foci further by explicitly focusing on racism rather than cultural or diversity sensitivity.

The review was thus guided by the following research questions: What is known from the existing literature about the experiences of racism by healthcare users and their caregivers in inpatient healthcare settings (hospitals and rehabilitation facilities) in high-income countries? Have intersectional perspectives been considered when describing these and if so, how?

### Stage 2: identifying relevant studies

**Fig. 1:**
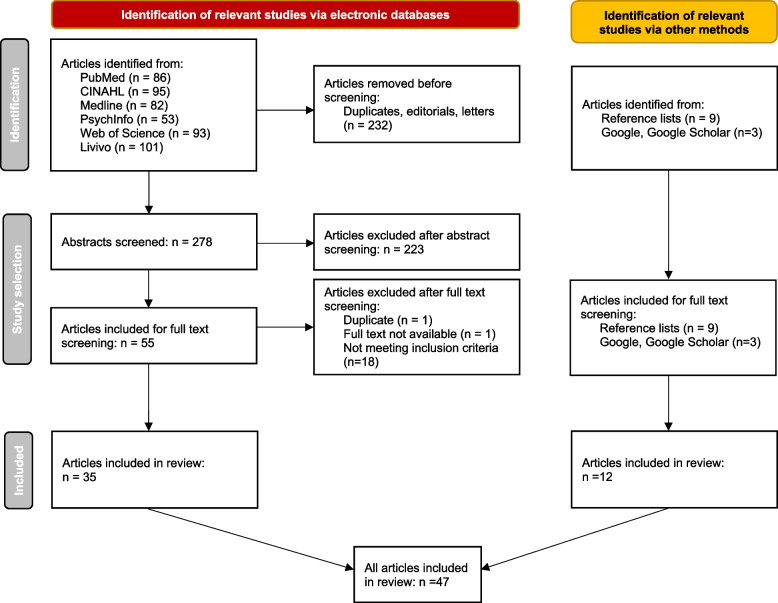
From: Page MJ, McKenzie JE, Bossuyt PM, Boutron I, Hoffmann TC, Mulrow CD, et al. The PRISMA 2020 statement: an updated guideline for reporting systematic reviews. BMJ 2021;372:n71. doi: 10.1136/bmj.n71. For more information, visit: http://www.prisma-statement.org/

A detailed search strategy that involved identifying evidence through electronic databases, reference lists and grey literature was developed (see Fig. 1). The search was limited to publications between 2013 and 2023; this was deemed most suitable for identifying the most current and relevant data.

### Electronic databases

We first searched relevant databases including Medline, PubMed, CINAHL, Livivo, PsycInfo and Web of Science based on the following inclusion criteria: publication date between 2013 and 2023; studies conducted in a high-income country using the World Bank Atlas method; and studies focusing on the experiences of healthcare users or their caregivers rather than professionals in inpatient care (hospitals or rehabilitation facilities). Papers presenting findings from original research with all study designs as well as reviews and meta-analyses were considered in both English and German. Comments, editorials and letters were excluded.

The following search string was developed for the PubMed database and later on adapted for each subsequent database, screening titles/abstracts:

 Search string: (Racism*) OR (racial discrimination) OR (race-based discrimination) OR (ethnic discrimination) OR (racial bias*) OR (racial stereotyp*) AND (healthcare) AND (clinic OR hospital* OR rehabilitation) AND (health work* OR profession* OR patient* OR family caregiver).

The database search was conducted by SM and regularly discussed with HY, AH and HTG. The identification of relevant studies was completed by 30 July 2023.

### Reference lists

In addition to the electronic databases, we searched the reference lists of all identified articles for further relevant studies that met our inclusion criteria. This step was crucial to ensure we did not neglect any relevant literature.

### Grey literature

Google Search and Google Scholar were used additionally in order to identify grey literature such as research reports, working papers, government documents or unpublished dissertations. While Google Scholar might have significant shortcomings for systematic reviews which limits its utility as a professional research tool [48, 49], it can be beneficial for the identification of grey literature [50]. We applied the same inclusion criteria as we did for the electronic database and reference list searches.

### Stage 3: study selection

All articles identified through the electronic database, reference lists and grey literature search that met our inclusion criteria were imported into Rayyan, an artificial intelligence-powered software developed to facilitate scientific reviews. Duplicates were deleted automatically or manually by SM where necessary. Title and abstract screening were conducted independently by SM and TA using Rayyan to avoid bias. All papers that met the inclusion criteria were stored separately in Rayyan for subsequent full-text screening. Any discrepancies between reviewers were resolved by consensus. Those papers who met the inclusion criteria underwent a secondary screening based on full texts by SM and TA with the support of HY, AH and JF.

### Stage 4: charting the data

Two researchers, SM and TA, independently extracted the data from the included studies into a Microsoft Word-based table. This involved a process of synthesising and sorting the studies according to their key characteristics (authors, source country, year of publication, publication type, theoretical approach, study design and methodology, aim of the study, important results and specifics of the inpatient setting). In a second step, any intersections of racism with other axes of discrimination addressed in the articles were also extracted.

### Stage 5: collating, summarising and reporting results

All results in tabular form as well as full-texts were uploaded into the software MAXQDA, developed for computer-assisted qualitative data analysis. All studies were read multiple times and key characteristics and themes were subsequently coded deductively and inductively using a thematic coding framework [51]. Coding aimed to capture both central characteristics as well as to map the specifics of racism in inpatient care and with respect to how intersectionality has been addressed by the included studies. Thematic codes required multiple rounds of refining and abstracting.

## Findings

An overview of the studies included in this review is provided in Table 1.

**Table 1:** Characteristics of included studies

No	Author(s), year, title	Setting	Objectives	Theoretical Framework(s)	Study design and methodology	Sample	Intersectional dimensions
*Peer-reviewed research articles*							
1	Akobirshoev, M. et al., 2019 [52]: Racial and ethnic disparities in birth outcomes and labour and delivery-related charges among women with intellectual and developmental disabilities	USA	To investigate racial and ethnic disparities in birth outcomes and labour and delivery-related charges among women with intellectual and developmental disabilities (IDD)	Health disparities, intersectionality	Logistic and linear regression	After weighting: estimated 10 308 delivery-associated hospitalisations of women with IDD, including 6228 delivery hospitalisations of White women with IDD, 2575 of Black women with IDD and 1505 of Hispanic women with IDD	Gender race, ethnicity, disability
2	Blakey et al., 2022 [53]: Effects of experienced discrimination in pediatric Sickle Cell Disease: Caregiver and provider perspectives	USA	To identify caregivers’ and providers’ perspectives on processes underlying discrimination and potential solutions to mitigate the negative effects of perceived discrimination among children with Sickle Cell Disease	Structural racism; social stigma	Questionnaires and semi-structured interviews guided by phenomenology	Caregivers (*N* = 27) of children with Sickle Cell Disease (≤ 12 years old) and providers from their hematology clinics (*N* = 11)	Race, motherhood
3	Capp et al., 2022 [54]: “They make you feel less of a human being”: Understanding and responding to Milwaukee’s racial disparity in infant mortality	USA	To understand how socioeconomic factors and racism create barriers to healthy pregnancies and birth outcomes for Black women, and identify practices necessary for overcoming these barriers	Lifecourse approach to health, intersectionality, ethnography	Ethnography (observations, interviews, focus groups)	N = 13: health professionals (n = 5); support group leader (n = 1); group leaders (n = 2); Mothers (n = 4)	Race, socio-economic status, gender
4	Askew, D.A. et al. 2021 [55]: “I’m outta here!”: a qualitative investigation into why Aboriginal and non-Aboriginal people self-discharge from hospitals	Australia	To understand why Aboriginal and non-Aboriginal people self-discharge from hospitals	None reported	Semi-structured interviews following a phenomenological approach	N = 11: Indigenous people (n = 5) and non-Indigenous people (n = 6) who had self-discharged	None reported
5	Hausmann et al., 2013 [38]: Perceived racial discrimination in health care and race differences in physician trust	USA	To understand whether two types of perceived racial discrimination, perceptions that the healthcare system is racially biased in general (perceived institutional racial discrimination) and perceptions that one has personally encountered racial discrimination while seeking health care (perceived interpersonal racial discrimination), mediated racial differences in patients’ trust in physicians	Perceived institutional racial discrimination, perceived interpersonal racial discrimination	Chi-squared tests and t tests, multiple mediator bootstrapping procedure	N = 430: Black people (n = 127); white people (n = 303)	None reported
6	Hemingway et al., 2021 [56]: Racial disparities in sustaining breastfeeding in a baby-friendly designated southeastern United States hospital: An opportunity to investigate systemic racism	USA	To assess if the introduction of BFHI changes racial disparities in breastfeeding practices	Racial disparities	Retrospective cohort study, chi-square and Fisher exact tests	N = 6,685	Race, gender, socio-economic status
7	Henry, R. et al., 2023 [57]: Disparities in care among gunshot victims: A nationwide analysis	USA	To examine racial differences in outcomes and resource utilization among victims of gunshot wounds in the United States	Racial disparities	Retrospective observational study, multivariable logistic regression	N = 250,675: White (n = 41,251), Black (n = 160,770), Hispanic (n = 46,484), Asian (n = 2,170)	None reported
8	Jacoby, S.F. et al., 2018: A safe haven for the injured? Urban trauma care at the intersection of healthcare, law enforcement, and race	USA	To describe how injured, Black patients perceived their interactions with police and what these perceptions reveal about police involvement within trauma care systems	Critical Race Theory	Mixed source, qualitative interviews	N = 24: male (n = 23), female (n = 1)	None reported
9	Petersen, J. et al., 2021 [58]: Ethnic inequalities in hospital admissions in England: an observational study	UK	To identify ethnic inequalities in inpatient hospital admission for all major disease categories in England	Health inequalities	Observational study, regression analyses	N = 40,928.105	Ethnicity, region
10	Janevic, T. et al., 2020 [59]: “Just because you have ears doesn’t mean you can hear”—perception of racial-ethnic discrimination during childbirth	USA	To investigate the impact of perceived racial-ethnic discrimination on patient-provider communication among Black and Latina women giving birth in a hospital setting	Critical Race Theory	Focus groups	N = 27: Black (n = 11), Latina (n = 16) women	Immigrant status, ethnicity, insurance status
11	Joseph, A., 2022: Racial and neighborhood disparities in mortality among hospitalized COVID-19 patients in the United States: An analysis of the CDC case surveillance database	USA	To characterize in-hospital mortality rates nationwide, determine whether in- hospital mortality for COVID-19 varied based on race and neighbourhood type, and evaluate for differences across census regions	Racial disparities	Chi-square and logistic regression analyses	N = 106,962: White (n = 55,468), Black (n = 22,589) Hispanic (n = 20,846) and Other (8,059)	Region, gender, socio-economic status
12	Joyes, E.C. et al., 2021 [60]: Inpatient institutional care: the forced social environment	UK	To highlight the problematic social environment of institutionalised mental healthcare, including the experience of racism	Interpersonal wellbeing	Ethnography, including participant observation and unstructured ethnographic interviews	300 h of participant observation with staff (n = 14) and residents (n = 14); interviews (n = 11)	Gender
13	Keshet, Y. and Popper-Giveon,, A. 2018 [61]: Patient demands for ethnic-based separation in public hospitals in Israel: patients’ and practitioners’ perspectives	Israel	To examine patients’ attitudes regarding separation between Jews and Arabs in inpatients rooms; to discover the coping strategies employed by healthcare practitioners	None reported	Mixed-methods: survey with healthcare users, in-depth interviews with staff	Survey: n = 760; interviews: n = 90	Ethnicity, religion and educational status
14	Worrall-Carter, L. et al., 2016 [62]: Exploring Aboriginal patients' experiences of cardiac care at a major metropolitan hospital in Melbourne	Australia	To explore Aboriginal patients’ lived experiences of cardiac care	Phenomenology	Open-ended, in-depth interviews	N = 10: male (n = 6), female (n = 4)	None reported
15	Tong, J. et al. 2022 [63]: Reporting of discrimination by health care consumers through online consumer reviews	USA	To examine how health care consumers perceive and report discrimination through public consumer reviews	Discrimination based on the Everyday Discrimination Scale	Review of online hospital reviews, qualitative coding	N = 10,535 reviews	None reported
16	Sperlich, M. and Gabriel, C. 2022 [64]: “I got to catch my own baby”: a qualitative study of out of hospital birth	USA	To investigate the Out-of –Hospital-Birth decision-making of two clinically important and understudied subgroups of women: Black women and women who have experienced childhood trauma	Trauma-sensitive care	In-depth qualitative interviews analysed using grounded theory	N = 18 interview participants	Gender
17	Solanki, J. et al. 2023 [65]: Experiences of adults from a black ethnic background detained as inpatients under the Mental Health Act (1983)	UK	To explore the experiences of people from a Black Ethnic background detained under the Mental Health Act	None reported	Semi-structured interviews analysed using a thematic coding framework	N = 12 interview participants (4 male, 8 female)	None reported
18	Shapiro, J. et al. 2022: Disposition decisions in cases of medical complexity and health inequity [66]	USA	To determine which undesirable options for discharge of a patient hospitalized with Covid-19 is most ethically permissible and to discuss actions to facilitate communication and decision- making in this situation	None reported	Case presentation	N = 1	Systemic racism, economic discrimination
19	Schmidt, I. et al. 2023: Patients’ perspectives on race and the use of race-based algorithms in clinical decision-making: a qualitative study [67]	USA	To examine patients’ perspectives on race and the use of race-based algorithms in clinical decision-making	None reported	Semi-structured interviews using a thematic and modified grounded theory approach for analysis	N = 23 interview participants	None reported
20	Schödwell, S. et al. 2022 [68]: Strukturelle Diskriminierung und Rassismus in der Krankenhausversorgung: die Rolle ökonomischer Rahmenbedingungen in der interkulturellen Öffnung	Germany	To identify challenges in the healthcare provision for people with a refugee or migration background, and subsequently address them through concrete measures	Racism without races (Balibar)	Guided interviews, thematic analysis	N = 112 interview participants	Religion, education, gender role expectations
21	Roder‐DeWan et al., 2021 [69]: Being known: A grounded theory study of the meaning of quality maternity care to People of Color in Boston	USA	To understand what high-quality maternity care means to people of color in Boston	Discrimination	Semi-structured interviews and focus groups analysed using a grounded theory approach	N = 23 interview participants	Interpersonal and structural racism
22	Priest, K.C. et al., 2022: Differences in the delivery of medications for opioid use disorder during hospitalization by racial categories: A retrospective cohort analysis [70]	USA	To understand differences in the delivery of buprenorphine versus methadone during acute medical or surgical hospitalizations for veterans with opioid use disorder (OUD) by racial categories (Black Non-Hispanic or Latino vs. White Non-Hispanic or Latino)	Racial differences/disparities	Retrospective analysis of electronic health record and administrative data from hospitalized adult veterans using logistic regression models	N = 1,313 unique patients	None reported
23	McGrath, C. et al. 2023: Identifying and mitigating disparities in central line– associated bloodstream infections in minoritized racial, ethnic, and language groups [71]	USA	To determine whether disparities in first central catheter–associated bloodstream infection (CLABSI) rates existed for pediatric patients of minoritized racial, ethnic, and language groups and to evaluate the outcomes associated with quality improvement initiatives for addressing these disparities	Racial disparities	Retrospective cohort study	N = 8,269	Languages other than English (LOE)
24	McLane, P. et al. 2021 [72]: First Nations members’ emergency department experiences in Alberta: a qualitative study	Canada	To better understand First Nations members’ ED experiences and expectations	None reported	Sharing circles; data was analysed using thematic analysis	N = 46	None reported
25	Mitchell, H. et al. 2020 [73]: Hospital outcomes for children with severe sepsis in the USA by race or ethnicity and insurance status: a population-based, retrospective cohort study	USA	To determine whether hospital outcomes in childhood severe sepsis were influenced by race or ethnicity and insurance status	Racial disparities	Retrospective cohort study using multilevel logistic regression performed on the 2016 database release from the Healthcare Cost and Utilization Project Kids’ Inpatient Database	N = 12,297	Ethnicity, insurance status
26	Mollard, E., Kupzyk, K. 2022 [74]: Birth satisfaction during the early months of the Covid-19 pandemic in the United States	USA	To describe birth satisfaction in women who gave birth in U.S. hospitals during the earliest months of the COVID-19 pandemic (March–July 2020)	None reported	cross-sectional survey using descriptive statistics, t-tests, analysis of variance (ANOVA) models, and nonparametric correlations	N = 747	None reported
27	Nelson, S., Hackman, H. 2013: Race matters: perceptions of race and racism in a sickle cell center [75]	USA	To identify perceptions of race and racism among both staff and patients/families with particular attention to provider attitudes as a potential contributor to racial healthcare disparities	Racism	Online survey, descriptive statistics	N = 247 (112 patients/families, 135 healthcare professionals)	None reported
28	Attanasio, L. and Hardeman, R., 2019 [76]: Declined care and discrimination during the childbirth hospitalization	USA	To investigate women's experiences of declining procedures in maternity care. Specifically, we examined the association between women's reports of declining medical procedures and perceived 29discrimination. Further, we assessed whether declining procedures was differently associated with perceived discrimination depending on the woman's race/ethnicity	Discrimination	Web-based survey, multivariate logistic regression model	N = 2,400	Gender
29	Phillipps-Beck, W. et al. 2020 [77]: Confronting racism within the Canadian healthcare system: Systemic exclusion of First Nations from quality and consistent care	Canada	To assess what is the root cause of racism against First Nation peoples in the healthcare system, what factors perpetuate racisms existence, what are the impacts of racism on First Nation health and what needs to be done to eradicate racism and to create an equitable healthcare system that sufficiently represents the needs, interests and values of First Nation peoples	Racism	Community-based and participatory research methods, analysis used principles of grounded theory, participant and Indigenous (decolonizing) research	N = 299 in-depth interviews, 8 focus group discussions	None reported
30	Vedam, S. et al., 2019 [78]: The Giving Voice to Mothers study: Inequity and mistreatment during pregnancy and childbirth in the United States	USA	To capture lived experiences of maternity care in diverse populations	Lived experience, phenomenology	Online cross-sectional survey, logistic regression	N = 2,138	Race, gender, mode of birth, place of birth, context of care
31	Eberly, L. et al., 2019 [79]: Identification of racial inequities in access to specialized inpatient heart failure care at an academic medical center	USA	To examine the relationship between race and admission service, and its effect on 30-day readmission and mortality	Inequities	Retrospective cohort study, multivariable generalized estimating equation models, Cox regression, propensity score analysis	N = 1967	Age, gender
32	Weber, T. et al., 2018 [80]: Leaving the emergency department without complete care: disparities in American Indian children	USA	To examine LWCET (leave the emergency department (ED) without complete evaluation or care) in American Indian children by exploring differences by ED location and utilization patterns	Racial disparities	Retrospective cohort study, univariate, multivariate and imputations analysis	N = 68,461 visits by 47,228 children	None reported
33	Lo, A. et al., 2018 [81]: A national study of U.S. Emergency Departments: Racial disparities in hospitalizations for heart failure	USA	To understand racial disparities in emergency department hospitalization patterns for heart failure and the factors that influence hospitalization	Racial disparities	Survey data, multivariable modified Poisson regression models	N = 12.2 million survey-weighted ED visits for heart failure	None reported
34	Qiao, W. et al. 2016 [82]: Relationship between racial disparities in ED wait times and illness severity	USA	To examine the hypothesis that, on average, black patients wait longer than nonblack patients and that the disparity is more pronounced as illness severity decreases	Racial disparities	Survey data, 2-model approach using natural logarithmic regression	N = 34,143 patient visits	Gender, age, insurance status, region, illness severity
35	McLemore, M. et al., 2018 [83]: Health care experiences of pregnant, birthing and postnatal women of color at risk for preterm birth	USA	To describe the pregnancy-related healthcare experiences of 54 women of color from Fresno, Oakland, and San Francisco, California, with social and/or medical risk factors for preterm birth	Racial disparities	Secondary analysis of focus group data using thematic analysis	N = 54	None reported
36	Fayfman, M. et al., 2016: Report on Racial disparities in hospitalized patients with hyperglycemia and diabetes [84]	USA	To determine the association between hyperglycemia, in patients with and without diabetes mellitus, and complications among different racial groups	Racial disparities	Observational study, using multiple logistic regression with sequential modeling	N = 35,866	Adjusted for age, gender, and BMI
37	Ali, I. et al., 2020 [85]: Racial disparities are present in the timing of radiographic assessment and surgical treatment of hip fractures	USA	To assess racial disparities in the care provided to patients with hip fractures	Racial disparities	Retrospective analysis of hospital records using multivariable generalized linear models	N = 1,535 (70% women)	None reported
*Reviews*							
38	Berg et al., 2019 [86]: Perspectives on Indigenous cultural competency and safety in Canadian hospital emergency departments: A scoping review	Canada	To identify and elaborate upon barriers and facilitators of cultural competency and safety in emergency departments in Canadian context	Cultural competency and safety; transcultural care	Scoping review based on methods outlined by the Joanna Briggs Institute	43 articles	Indigeneity, socio-economic status
39	Chen et al., 2021: Racial/Ethnic inequities in healthcare‑associated infections under the shadow of structural racism: narrative review and call to action [87]	USA	To review racial and ethnic inequities in the incidence and prevention of healthcare-associated infections in the USA, identify gaps in the literature, and recommend future directions to mitigate these inequities	Structural racism; health inequities	Narrative review	None reported	None reported
40	Espiner et al., 2021 [88]: Barriers and facilitators for Māori in accessing hospital services in Aotearoa New Zealand	Aotearoa/ New Zealand	To understand the barriers and facilitators of access to hospital services for Māori	Health inequities; racism	Literature review	23 articles	None reported
41	Graham, R. et al., 2020 [89]: Experiences of Māori of Aotearoa New Zealand’s public health system: A systematic review of two decades of published qualitative research	Aotearoa/New Zealand	To synthesise the broader perspectives of Māori patients and their whānau (extended family, family group) of their treatment within the public health system	Critical community psychology approach	Systematic reviews	14 articles	None reported
42	Sim, W. et al. 2021: The perspectives of health professionals and patients on racism in healthcare: A qualitative systematic review	USA, UK, Australia, Canada, Spain, Hong Kong	To understand racial bias in clinical settings from the perspectives of minority patients and healthcare providers to inspire changes in the way healthcare providers interact with their patients	Racial bias	Thematic synthesis using the Thomas and Harden framework	23 articles	None reported
43	Sanjida, S. et al. 2022 [90]: Indigenous Australians’ experiences of cancer care: A narrative literature review	Australia	To show current evidence about the experiences of Indigenous people with cancer care services in Australia’s primary and tertiary healthcare systems	None reported	Narrative literature review	23 articles	None reported
44	DiMeglio, M. et al. 2018: Factors underlying racial disparities in sepsis management [91]	USA	To understand what is known about factors driving racial disparities in sepsis and to suggest potential interventions aimed at reducing health disparities in the prevention, early identification, and clinical management of sepsis	Racial disparities	Focused review	unclear	Socio-economic status, insurance status, gender
45	McGowan, S. et al. 2021 [92]: Racial disparities in ICU outcomes: A systematic review	USA	To systematically analyse the literature to assess the prevalence of racial disparities in the ICU	Racial disparities	Systematic review	N = 25 articles	None reported
46	Watson, H., Downe, S. 2017 [93]: Discrimination against childbearing Romani women in maternity care in Europe: a mixed-methods systematic review	Europe (countries belonging to the Council of Europe)	To review the published evidence on discrimination against Romani women in maternity care in Europe, and on interventions to address this	Racial discrimination	Mixed methods systematic review	N = 10	None reported
*Grey literature*							
47	Center for Reproductive Rights, 2014 [94]: Reproductive Injustice: Racial and Gender Discrimination in U.S. Health Care. A Shadow Report for the UN Committee on the Elimination of Racial Discrimination	USA	To fill the gaps in the U.S. government’s report on the status of women’s rights to substantive equality, non-discrimination, and other core human rights protected by the ICERD	Racial and gender discrimination	Narrative interviews	None reported	Citizen status, gender, race, ethnicity

### Descriptive findings

Overall, we included 47 articles for analysis (see Table 1). Thirty-five were identified through the electronic database search, and 12 through Google, Google Scholar and by a manual screening of the reference lists of included studies. Eighteen articles (38%) were published before 2020 while 29 (62%) were published in or after 2020. Their countries of origin can be found in Table 2.

**Table 2:** Countries of origin

Country of origin	No. of studies
USA	32
Canada	3
Australia	3
UK	3
New Zealand/Aotearoa	2
Israel	1
Germany	1
Multi-country	2
**Total**	**47**

Articles from the US context analysed the experiences of Black or Latinx healthcare users or of people of colour in general, though racial classifications were not used consistently [38, 52–54, 56, 57, 59, 63, 64, 66, 67, 69–71, 73–76, 78–85, 87, 91, 92, 94]. One study examined the experiences of American Indian children [80]. Articles from the Australian, New Zealand/Aoteroa and Canadian contexts analysed the experiences of and barriers faced by indigenous groups, i.e. Māori, indigenous Australians and First Nations respectively [55, 62, 72, 77, 86, 88–90]. One study from Europe analysed the experiences of Romani women across national contexts [93], while the only German study we found focused on migrants and refugees [68]. The single article we identified from Israel focused on religious/ethnic tensions between Jewish and Arab Israelis [61]. The majority of the articles focused on the hospital setting, either exclusively or as part of an analysis of the overall healthcare system. One article used the hospital system to compare out-of-hospital experiences in a birthing centre [64]. No articles analysed a rehabilitation setting or explicitly addressed the experiences of (racialised) caregivers though one [53] considered their perspectives.

Research on reproductive services predominantly examined the overall experiences of maternity care by people of colour [69, 94], especially those at risk of pre-term birth [83], as well as Romani women [93]. Other articles analysed disparities in birth outcomes among women of colour with intellectual and developmental disabilities [52], infant mortality [54], birth satisfaction during COVID-19 [74] and breastfeeding practices in a baby-friendly hospital [56]. Various articles focused on racial discrimination during childbirth [59, 64, 76, 78], leading some women to choose an out-of-hospital, community-based birth for their subsequent births [64]. Studies from the field of paediatrics varied in focus; one study examined the effects of perceived racial discrimination on caregiver-provider interactions in a sickle cell disease centre by both staff and carers [53]; others analysed the hospital outcomes of racialised children with severe sepsis in the US [73] as well as decisions for early discharge and for leaving the emergency department without complete evaluation and treatment (LWCET) [80]. In emergency care, included articles analysed people’s experiences with emergency ward visits more broadly [72] as well as the reasons for the decision to leave the emergency ward without complete treatment [80]. Others examined disparities in hospitalisation for racialised healthcare users presenting to the emergency ward with heart failure [81] and the relationship between racial disparities in emergency ward wait times and illness severity [82]. The one study examining intensive care was a review investigating racial disparities in intensive care unit (ICU) outcomes overall [92]. Studies examining the hospital system overall predominantly focused on ethnic inequalities in hospital admissions [58], discrimination during hospital stays [68] and overall interactions between hospital staff and racialised groups [89]. Studies examining mental health focused exclusively on forensic psychiatry [60, 65].

### Thematic findings

#### Conceptual approaches

Four main thematic or conceptual approaches related to healthcare users’ experiences with (racial) discrimination have been used in the articles we identified. First, some articles studied the experiences of specific racialised populations in interaction with the healthcare system, sometimes using a phenomenological or life course approach. Graham et al. [89] and Espiner et al. [88], for instance, have examined the overall experiences of Māori in the New Zealand/Aotearoa public health system, including barriers in accessing care. In another instance, Roder-DeWan et al. [69] sought to understand what high-quality maternity care means to women of colour in Boston. Here, racism was one of a plethora of experiences that minoritised healthcare users faced when using healthcare services. Second, most quantitative studies used a disparities approach that described existing racial disparities, for instance in breastfeeding practices [56], gunshot victims [57] or ICU outcomes [92] but did not engage more deeply with the structural origins of these disparities. This is in line with other studies revealing that racial health disparities literature rarely embeds race and racism in their social and historical contexts to explain relational aspects of racial inequity [95]. Third, while all articles suggested, albeit sometimes vaguely, racism to be a key driver of existing disparities, only some studies have explicitly focused on racism or racial discrimination as their main object of investigation. For example, Janevic et al. [59], Sperlich and Gabriel [64], Attanasio et al. [76] and Vedam et al. [78] have all analysed perceived racial discrimination during childbirth, whereas Phillips-Beck et al. [77] have examined the systemic racism and exclusion of First Nations from quality healthcare in Canada. A fourth approached evolved around concepts of cultural competency and safety, for example in emergency wards in Canada [72, 86]. Racism did play a role as one of the drivers of adverse experiences but the conceptual framework of these articles was derived from studies around cultural competency.

#### Specifics of racism in inpatient care

While all articles discussed encounters of racism in the healthcare system, some stressed how racism manifests and operates specifically in an inpatient setting, and what effects these specifics of the inpatient setting have on the care available to racialised healthcare users.

Overall, racialised groups were reported to have lower hospitalisation, admission and referral rates within and across hospitals than non-racialised groups. For instance, Lo et al. [81] found that among all non-ICU admissions, Black patients were less likely to be hospitalised than white patients when aged 65+ and needing care more acutely. Black patients with sepsis in an ICU who were mechanically ventilated were also found to be less likely to be transferred to a higher level of care [92]. Similar findings have led Eberly at al. [79] to argue that lower admission rates for racialised patients with heart failure are a key intra-hospital driver of racial inequity. Racialised patients have also been reported to have longer wait times to admission or referral. Qiao et al. [82] found that, on average, Black patients in the US have significantly longer mean wait times in the emergency ward than white patients, and this increased as illness severity decreased. No racial disparities in wait times were found for critically ill patients though. However, one article found that Black and Latinx children with severe sepsis had longer hospital stays than white children; when death was accounted for as a competing risk, Black as well as Latinx children had a markedly reduced probability to be discharged from hospital alive by day 30 [73]. This correlates with the overall higher mortality rate for Black children with severe sepsis and is, according to the authors, at least partly attributed to the differences in the quality of care received in hospitals frequented by racial or ethnic minorities, testament to structural racism (ibid).

Another manifestation of racism in acute, inpatient care is the spatial segregation of patients according to ethnic and religious [61] or racialised criteria [93]. Segregation has been reported between Arab and Jewish Israeli patients in an Israeli hospital, predominantly due to Jewish patients’ demands, and nurses’ attempts to prevent unnecessary tensions [61]. The authors note that in some cases this constitutes discrimination against Arab patients. Similarly, Watson et al. 2017 [93] surveyed the widespread evidence of race-based segregation of Romani women on maternity wards across Europe (defined as those countries belonging to the Council of Europe). Not only are Romani patients frequently being separated from white patients, but ‘Romani’ rooms have also been described as being of poorer quality, not being cleaned by hospital staff, lacking heating and containing fewer facilities such as toilets [93]. Hospital staff frequently justified these practices on the basis that it was to protect Romani women from the racism of white women and their families, but the analysis also illustrated healthcare providers’ pervasive bias about Romani women’s lifestyles and behaviour patterns. Some staff also argued that separation was necessary for hygienic reasons or to protect non-Roma white women, demonstrating the extent of racist practices Romani women are exposed to when giving birth in a European hospital.

Last, one study stressed the additional threat of racist abuse by fellow healthcare users in inpatient care [60]. Residents of an inpatient forensic mental health hospital in the UK reported of having been verbally attacked by other residents, leading to a heightened sense of vigilance and the avoidance of communal areas. Bullying and even threats of physical violence were other forms of abuse reported by residents. While not discussed in much detail, threats of discrimination by other healthcare users are also eminent in the attempt to segregate these users along racialised lines [61, 93].

#### Intersectional dimensions considered

The majority of the articles did not explicitly focus on intersections of race or racism with other social categories such as gender or age. While many described the social stratification of the sample composition, they did not specify how experiences of racism were shaped by these interactions. For instance, Worrall-Carter et al. [62] reported the gender of their respondents but did not examine whether it influences indigenous Australians’ experiences of cardiac care. Social categories used for describing the intersections of race included predominantly gender, socioeconomic status or proxies such as educational attainment (e.g. [61]). Some also analysed race in interaction with insurance status [73], immigration status [59], religion [61], age [79], and intellectual and developmental disabilities [52]. Quantitative studies used social categories such as gender as covariates or adjusted for gender in regression models (e.g. [52]).

Only one qualitative study explicitly used an intersectional framework: Capp et al. [54] examined the intersections of socioeconomic deprivation and racism and how they affect barriers to healthy pregnancies and birth outcomes in Milwaukee through the lens of Black mothers and health professionals. Their analysis illustrated that the entanglements of public apathy, violence and stress, discrimination and mistrust shape the experience of Black women, and especially Black mothers, in the healthcare system. While not drawing explicitly on an intersectional framework, Berg et al. [86], in their review of barriers and facilitators of cultural competency and safety in emergency wards in Canada, have also found that patients expressed fear of discrimination more often when they belonged to more than one marginalised group. For example, indigenous patients who also belonged to lower socio-economic strata and experienced addictions expressed particular concerns about the discriminating practices of healthcare providers.

### Effects and coping strategies

A range of effects and coping strategies for these dynamics of racism in the inpatient sector have been described in the literature. In particular, experiences of racism in healthcare have been found to lead to avoidance tactics and withdrawal from the healthcare system. Withdrawal can be the result of racist discrimination by healthcare staff but also by other residents. Joyes et al. [60] found that the threat of racist abuse in a forensic psychiatric institution can lead to the avoidance of group therapy sessions or other community events. This not only minimised bullied healthcare users’ opportunities for social interaction and connectedness but also posed a risk to their mental health.

Racialised healthcare users have been found to actively decline care due to discrimination [76] to the extent that they prematurely discharge from the hospital [55, 80, 83]. Women of colour at risk for preterm birth who felt neglected and offered poorer quality care have been found to independently leave the hospital in search for better quality care elsewhere [83]. American Indian children attending emergency departments have also been found to discharge or leave without complete evaluation and treatment more frequently than white children [80]. Amongst a cohort of paediatric patients in the US Midwest, an overall 1,7% of paediatric visits resulted in leaving without complete evaluation and treatment (LWCET); of those, American Indian children had much higher odds of LWCET than white children. Another study investigating why indigenous Australian patients self-discharge from hospital described a ‘tipping point’, the cumulative impact of unmet needs, which was compounded by experiences of interpersonal and institutional racism [55]. While it is commonly assumed that people who self-discharge do not care about their health, the authors found that the opposite was the case for their study participants: not only was their self-discharge a rational act of reclaiming their personhood and agency but for self-discharging indigenous people, it was also an attempt to *prioritise* their health and wellbeing.

Those who avoided public hospitals often sought care in more community-based settings such as First Nation Home Care [77] or sought the assistance of community liaison people inside public hospitals [72]. The latter work to improve coordination between community health organisations and hospitals, especially around discharge planning and management.

## Discussion

To our knowledge, this is the first scoping review that explored the specifics of racism in inpatient care in several high-income countries, and focused on the consideration and operationalisation of intersectionality. While US-American studies continue to set the terms of discussion, not least due to the general availability of data on race and ethnicity, other high-income countries, including Canada, Australia, New Zealand and the UK, also examine racism in their own healthcare systems. Nationally-specific understandings of racism and priorities vis-à-vis underserved communities are evident in the research; for instance, in Canada, Australia and New Zealand most articles examined the experiences of indigenous communities and their barriers to accessing quality care. For articles from the US, a key concern was the racial disparities between Black and white Americans (as well as, to a lesser extent, Latinx patients), representing the key racialised tensions in the country. In Germany, where racial data is not routinely collected and an awareness of indigenous, non-immigrant racialised groups is only evolving [96], the one article we found focused on the experiences of recent immigrants and refugees.

Absent from the literature were studies from other European countries as well as studies focussing on a wider variety of racialised and disadvantaged groups, especially native born, non-immigrants. The former is surprising as research on racism in healthcare in the Nordic countries such as Sweden [3, 97–99] is growing rapidly. However, other European countries also exhibit stark disparities in healthcare access and utilisation between different social groups, and especially between recent immigrants and naturalised citizens [100]. This is testament to the continued “absent presence” of race in Europe [101]. Here, the post-war rejection of race as a scientific category simultaneously rendered the discussion of racism a highly sensitive, and often ignored, endeavour. Instead, research and social policy have predominantly focused on issues around migration and the ‘integration’ of recent immigrants. The most immediate consequence of this paradigm is a lack of evidence on racism, including in healthcare, in Europe more broadly. Moreover, the sparse evidence that does exist is drawn from studies on forced migrants and refugees. Native populations who are also racialised are rarely examined, limiting the knowledge and understanding of nationally-specific dynamics of racism in European healthcare.

In the articles we identified, intersectional dimensions were only considered to a limited extent, though of course this may also be due to the precise search terms used for this review. Research employing similar concepts such as misogynoir (e.g. [102]) or focusing on the discrimination of two-spirit people in healthcare (e.g. [103]) could not be identified. This means that our analysis is limited to those articles that explicitly used an intersectional approach and may have missed important other perspectives. While quantitative studies usually used several variables to adjust for different racial groups, adjustment does not fully comply with an intersectional framework that postulates the unique experiences produced by intersecting axes of oppression in a non-additive way [104–106]. Moreover, intersectionality necessitates not only the analysis of intersecting identities but also of processes and structural forms of discrimination in order to understand the macro-level drivers of inequity [107]. Intersectionality scholarship in public health has recently been gaining traction, and a range of new methods and techniques to operationalise an intersectional framework have been developed [104, 108–110]. Research examining racism in the healthcare sector would benefit from an engagement with this literature to produce a more detailed and comprehensive understanding of the effects of interactions of racism with other axes of discrimination. This would also allow for more targeted interventions tackling racism in healthcare.

While we identified a range of different articles examining experiences of racism in inpatient care, few of them delved deeper into the specifics of this setting. This may not be surprising as healthcare users may not differentiate between inpatient and outpatient settings when describing their experiences. Our review of the published literature has shown, however, that inpatient settings exhibit a range of specific structures and conditions that produce unique experiences and forms of racism, including varying admission, hospitalisation and referral rates, spatial segregation, and the threat by other healthcare users. As such, future research ought to examine these structures in more detail in order to gain a more comprehensive understanding of how racism operates in the inpatient setting.

This is also imperative in order to eventually develop more embedded and targeted anti-racist interventions for health policy-making and practice. Hassen et al. [19] have suggested six key requirements for any such intervention to be successful: an explicit, shared language of anti-racism; a thorough commitment on the side of the institutional leadership; a multi-level approach that includes policy and organisational interventions; transparency and accountability mechanisms; long-term and meaningful partnerships with racialised communities; and the investment in ongoing and mandatory anti-racist education and training. Based on our review, we suggest two additional, specific interventions for the inpatient care sector, following the tenets of everyday racism encompassing both interactional and structural-cultural forms of racism. First, to counter the implicit bias in non-clinical decision-making over room allocation, wait times and the spatial division of patients, any anti-racist intervention such as training for staff aiming to promote anti-racism must not only be offered to medical and nursing professionals but also include support staff such as receptionists and nursing aides. Any such training should include the critical reflection of how the staff’s own positionalities, including positionalities shaped by privilege, affect their interaction with racialised healthcare users. Targeted to the specific healthcare institution, such trainings can help develop a deeper understanding of different forms of racism prevalent in the respective individuals’ working contexts. Moreover, as limited time and a stressful working environment can accelerate the use of prejudices and stereotypes at all levels, better overall working conditions are crucial to reducing racial discrimination. Second, existing research on racism as experienced by healthcare users has largely focused on staff, clinical and non-clinical, as perpetrators of this racism. However, this review has demonstrated that fellow patients can be another source of experiences of racism leading to the further alienation of racialised healthcare users from the healthcare system and exacerbate health inequities as these users withdraw from therapy or treatment. Future anti-racist interventions must therefore also target the threat of interpersonal racism emanating from a range of actors in the healthcare system beyond the focus on professional staff. A robust and low-threshold complaints structure or ombudsman for discrimination should be structurally anchored at the institutional level, ideally offering multilingual counselling and advice. Such services can provide immediate support, engage in advocacy and point the person concerned towards the next steps. It is important that these services are located on the premises of the hospital or rehabilitation centre, ideally centrally located and easily accessible, as patients treated there may have longer stays and impaired mobility, preventing them from seeking advice elsewhere. To build trust, ensure continuous support and strengthen ties with affected communities, these services should also be linked to existing advocacy and community organizations.

### Limitations

Our review was limited to articles in English and German, and only focused on high-income countries. Accounts of racist discrimination in low- and middle-income countries, which may also have produced important evidence on racism in inpatient care, have been excluded to increase the feasibility of this review. Moreover, while we aimed to consider the hospital and rehabilitation sector as an understudied area of healthcare delivery, our review did not actually yield articles analysing the rehabilitation sector. Racism in rehabilitation centres therefore constitutes an important future area of research.

## Conclusion

This scoping review has examined the existing literature on racism in inpatient care (hospitals and rehabilitation centres) in high-income countries, with a specific focus on how intersectional dimensions have been taken into consideration. Analysing 47 articles, it identified three main specifics of racism in inpatient care: varying hospitalisation, admission and referral rates; the spatial segregation of healthcare users along racialised criteria, and discrimination by fellow healthcare users. As such, this review has illuminated an understudied phenomenon with important implications not only for future research but also health policy-making and practice.

Intersectional dimensions were rarely taken into account; while the majority of papers provided a demographic description of study samples, only one study used an explicitly intersectional framework focusing not only on the unique effects of intersecting axes of discrimination but also on their structural drivers. Here, too, this review has shown that more in-depth research is needed on the intersectional entanglements of racism in inpatient care.

## Data Availability

All data generated or analysed during this study are included in the supplementary information files of this article.
